# Synthetic Spectrum Approach for Brillouin Optical Time-Domain Reflectometry

**DOI:** 10.3390/s140304731

**Published:** 2014-03-07

**Authors:** Ken'ichi Nishiguchi, Che-Hsien Li, Artur Guzik, Kinzo Kishida

**Affiliations:** 1 Office for University-Industry Collaboration, Osaka University, Yamadaoka 2-8, Osaka 565-0871, Japan; 2 Neubrex Co., Ltd., Sakaemachi-dori 1-1-24, Kobe 650-0023, Japan; E-Mails: Li-z@neubrex.jp (C.-H.L.); guzik@neubrex.jp (A.G.); kishida@neubrex.jp (K.K.)

**Keywords:** Brillouin scattering, distributed sensor, optical fiber sensor, optical time-domain reflectometry (OTDR), strain measurement, synthetic spectrum, temperature measurement

## Abstract

We propose a novel method to improve the spatial resolution of Brillouin optical time-domain reflectometry (BOTDR), referred to as synthetic BOTDR (S-BOTDR), and experimentally verify the resolution improvements. Due to the uncertainty relation between position and frequency, the spatial resolution of a conventional BOTDR system has been limited to about one meter. In S-BOTDR, a synthetic spectrum is obtained by combining four Brillouin spectrums measured with different composite pump lights and different composite low-pass filters. We mathematically show that the resolution limit, in principle, for conventional BOTDR can be surpassed by S-BOTDR and experimentally prove that S-BOTDR attained a 10-cm spatial resolution. To the best of our knowledge, this has never been achieved or reported.

## 1. Introduction

The spectrum of Brillouin scattering in optical fiber shifts in proportion to changes in the strain and temperature of the fiber. Brillouin optical time-domain analysis (BOTDA) [1,2] and Brillouin optical time-domain reflectometry (BOTDR) [3,4] are distributed measurement techniques utilizing this property by injecting a pulsed pump light and observing the scattered light. The amount of strain/temperature change is estimated by measuring the spectral shift, while its position is determined by the round-trip time of the light. Since BOTDR uses only one end of a fiber, it is suitable for long distance measurement, whereas BOTDA uses pump and probe lights that are injected from both ends of a fiber.

To improve the spatial resolution of both techniques, it is necessary to narrow the pulse; however, the linewidth of the observed spectrum then becomes wider and makes measurement of the spectral shift difficult. For this reason, the spatial resolution has been limited to about one meter in both techniques [5,6].

For BOTDA, Bao *et al.* [7] found experimentally a phenomenon in which the spectral linewidth becomes shorter when a very short pulse of about 1 ns is used. This phenomenon arises only when there is light leakage. Inspired by this discovery, various resolution improvement methods have been proposed [8–14]. The pump light of BOTDA plays two roles: phonon excitation and scattering by the phonons. The width of the pump light must be longer than the phonon lifetime (about 9 ns) for phonon excitation, whereas it must be much shorter than 10 ns, which corresponds to a one-meter resolution, for high spatial resolution. To satisfy these incompatible requirements, an idea to construct a pump light using a long and a short element was born. The long element, which is a long pulse or a CWwave, takes the role of phonon excitation, and the short element takes the role of being scattered by the phonons. A part of the spectrum is by the combination of these elements. By emphasizing this desired part, high-resolution measurement could be attained.

For the construction of pump light from two elements, the authors of [8–11] used amplitude modulation and [12,14] used phase modulation. In any construction method, the measured spectrum includes both desired and undesired parts. Parameters must be optimized to emphasize only the desired part. Instead of that, the authors of [12–14] proposed methods to suppress or cancel the undesired part of the spectrum by combining two different measurements. By these methods, high-resolution measurement of centimeter-order for BOTDA has been attained.

On the other hand, the pump light of BOTDR plays a single role, since the BOTDR utilizes spontaneous scattering, so that it is difficult to attain high resolution by the same idea as for BOTDA. For resolution improvement of BOTDR, Koyamada *et al.* [15] proposed a double pulse method that yields an oscillatory Brillouin spectrum and verified 20-cm spatial resolution by experiment.

In this paper, we propose a novel method, referred to as synthetic BOTDR (S-BOTDR), to improve the spatial resolution of BOTDR. In this method, a synthetic Brillouin spectrum is constructed by combining several spectrums obtained by BOTDR measurements with different pump lights and low-pass filters. The pump lights and low-pass filters are composed of short and long elements with phase differences. We mathematically show that the resolution limit in principle for conventional BOTDR could be overcome by S-BOTDR and experimentally prove that 10-cm spatial resolution, which is much smaller than the conventional BOTDR limit, is attained by S-BOTDR.

The remainder of this paper is organized as follows: Section 2 presents the mathematical formulation of BOTDR and its performance limit in principle. In Section 3, the proposed method, called S-BOTDR, is described. Sections 4 and 5 are devoted to the evaluation of the proposed method through simulations and experiments, respectively. Finally, we conclude our paper in Section 6.

## 2. Mathematical Model of BOTDR

### 2.1. Sensing Mechanism of BOTDR

**Figure 1.**
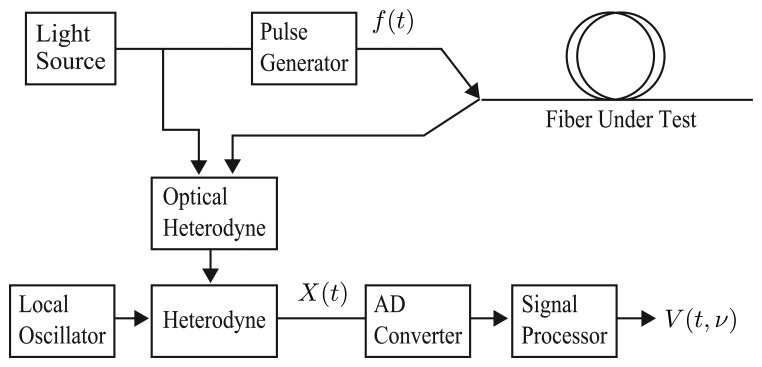
Block diagram of Brillouin optical time-domain reflectometry (BOTDR).

A BOTDR system is shown in Figure 1. The light from the light source is divided into a pump pulse and a reference continuous wave. In an optical fiber, the acoustic waves are always excited by thermal fluctuation and the pump pulse is scattered by the acoustic waves in all parts of the fiber. Since the acoustic waves move with the sound velocity of the medium, the backscattered light has a Doppler shift, which is called a Brillouin frequency shift (BFS). As the sound velocity changes in proportion to the change in strain or temperature in each position of the fiber, the BFS also changes in proportion to them. In BOTDR, the backscattered light is heterodyned with the reference wave and the spectrum of the interfering light; *i.e.*, the Brillouin spectrum is obtained. The BFS is estimated from the Brillouin spectrum as its center frequency. Since the round-trip time of the light corresponds to the position in the fiber, the BFS at each position in the fiber is obtained. Thus, distributed sensing of strain and temperature is possible using the Brillouin scattered light.

### 2.2. Basic Equations of BOTDR

Brillouin scattering in optical fiber is described by the following equations [16–18]:

(1) $$\left(\frac{1}{\upsilon_{\text{g}}}\frac{\partial }{\partial t}+\frac{\partial }{\partial z}+\frac{\alpha }{2}\right)\;E_{\text{p}}=i\kappa \rho E_{\text{s}}$$

(2) $$\left(\frac{1}{\upsilon_{\text{g}}}\frac{\partial }{\partial t}-\frac{\partial }{\partial z}+\frac{\alpha }{2}\right)\;E_{\text{s}}=i\kappa \rho *E_{\text{p}}$$

(3) $$\frac{\partial \rho }{\partial t}+\left(\frac{\Gamma_{\text{B}}}{2}+2\pi i\nu_{\text{B}}(z)\right)\;\rho =i\wedge E_{\text{p}}E_{\text{s}}^{*}+R(z,t)$$

where *E*_p_, *E*_s_ and *ρ* are complex amplitudes of a pump light, a backscattered light and an acoustic wave, respectively, *κ* and Λ are coupling coefficients, *υ*_g_ is the light speed in the fiber, *α* is the fiber loss factor and superscript * denotes a complex conjugate. Γ_B_ is the linewidth of the acoustic wave spectrum and *ν*_B_(*z*) denotes the BFS at position *z*. Here, the spectral linewidth is defined by the full width at half maximum.

Random force *R*(*z, t*) is a circular symmetric complex white noise that is white both in space and time; *i.e.*, it is characterized by *E* [*R*(*z*, *t*)R*(*z′*, *t′*)] = *Qδ*(*z*−*z′*)*δ*(*t*−*t′*), where *E*[·] stands for expectation and *Q* is a constant. The two terms in the right-hand side (RHS) of Equation (3) correspond to stimulated and spontaneous Brillouin scattering, respectively Under usual BOTDR conditions, as analyzed in a previous paper [19], the largest part of the measured spectrum arises from spontaneous scattering, and the stimulated scattering term can be neglected. Moreover, then, since it becomes |*E*_s_| ≪ |*E*_p_|, the RHS of Equation (1) can be neglected. Therefore, BOTDR is described by the following equations:

(4) $$\left(\frac{1}{\upsilon_{\text{g}}}\frac{\partial }{\partial t}+\frac{\partial }{\partial z}+\frac{\alpha }{2}\right)\;E_{\text{p}}=0$$

(5) $$\left(\frac{1}{\upsilon_{\text{g}}}\frac{\partial }{\partial t}-\frac{\partial }{\partial z}+\frac{\alpha }{2}\right)\;E_{\text{s}}=i\kappa \rho *E_{\text{p}}$$

(6) $$\frac{\partial \rho }{\partial t}+\left(\Gamma +2\pi i\nu_{\text{B}}(z)\right)\;\rho =R(z,t)$$

The boundary conditions of *E*_p_(*z, t*) and *E*_s_(*z, t*) are given by:

(7) $$E_{\text{p}}(0,t)=\sqrt{\frac{P_{\text{p}}}{A_{\text{eff}}}}f(t)$$

(8) $$E_{s}\;\left(z,\frac{z}{\upsilon_{g}}\right)=0$$

where *P*_p_ and *f*(*t*) denote the power and shape function of the pump pulse injected into an optical fiber, respectively, *A*_eff_ is the effective core area of a fiber and Γ = Γ_B_/2 is set.

### 2.3. Analytical Solution to the BOTDR Equations

The solution to last section's BOTDR equations can be represented analytically. For simplicity, assuming that the fiber loss is small, we set *α* = 0.

First, the solution to Equation (1) under boundary Condition Equation (7) is represented as:

(9) $$E_{\text{p}}(z,t)=\sqrt{\frac{P_{\text{p}}}{A_{\text{eff}}}}f\;\left(t-\frac{z}{\upsilon_{g}}\right)$$

Next, the stationary solution to Equation (6) is represented as:

(10) $$\rho (z,t)=\int_{-\infty }^{t}e^{-(\Gamma +2\pi i\nu_{B}(z))(t-s)}R(z,s)ds$$

whose autocorrelation function is given by:

(11) $$E[\rho (z,t)\rho^{*}(z^{\prime },t^{\prime })]=\frac{Q}{2\Gamma }\delta (z-z^{\prime })e^{-2\pi i\nu_{\text{B}}(z)(t-t^{\prime })}e^{-\Gamma |t-t^{\prime }|}$$

Then, substituting (9) and (10) into (5) and solving it under Condition (8), we obtain:

(12) $$E_{s}(z,t)=i\kappa_{1}\int_{z}^{L_{f}}f\;\left(t-\frac{2z^{\prime }-z}{\upsilon_{\text{g}}}\right)\;\rho^{*}\;\left(z^{\prime },t-\frac{z^{\prime }-z}{\upsilon_{\text{g}}}\right)dz^{\prime }$$

where $\kappa_{1}=\sqrt{P_{\text{p}}/A_{\text{eff}}}\kappa$ and *L*_f_ is the length of the fiber.

The backscattered light returned to the input end of an optical fiber in BOTDR is represented as:

(13) $$X(t)\overset{\text{def}}{=}E_{s}(0,t)=i\kappa_{1}\int_{0}^{L_{\text{f}}}f\;(t-\frac{2z}{\upsilon_{\text{g}}})\;\zeta^{*}(z,t)dz$$

where we set *ζ*(*z*, *t*) ≡ *ρ*(*z*, *t* − *z*/*υ_g_*), which has the same statistical property as *ρ*(*z, t*); *i.e.*,

(14) $$E[\zeta (z,t)\zeta^{*}(z^{\prime },t^{\prime })]=\frac{Q}{2\Gamma }\delta (z-z^{\prime })e^{-2\pi i\nu_{\text{B}}(z)(t-t^{\prime })}e^{-\Gamma |t-t^{\prime }|}$$

holds. We note that *X*(*t*) becomes a circular complex Gaussian (ccG) process with mean zero. The component of this signal with frequency *ν* is obtained by:

(15) $$\begin{array}{ll} Y(t,\nu ) & =c_{Y}h(t)*[X(t)e^{-2\pi i\nu t}]\\ & =i\kappa_{2}\int_{-\infty }^{\infty }h(t-\tau )e^{-2\pi i\nu \tau }\int_{0}^{L_{\text{f}}}f\left(\tau -\frac{2z}{\upsilon_{g}}\right)\zeta *(z,\tau )\text{dzd}\tau \end{array}$$

where *c_Y_* is a constant, *h*(*t*) is an impulse response of a low-pass filter and * denotes a convolution, and we set *κ*_2_ = *c_Y_ κ*_1_. *Y*(*t, ν*) also becomes a ccG process with a mean of zero as *X*(*t*).

### 2.4. Brillouin Spectrum and Point Spread Function

The Brillouin spectrum obtained by one measurement of BOTDR is represented as:

(16) $$V(t,\nu )={\left|Y(t,\nu )\right|}^{2}$$

Since BOTDR's signal source is thermally excited acoustic waves, its spectrum fluctuates even without observation noise. Although the spectrum is averaged by many replicate measurements, we must suppress the fluctuation for one measurement.

Since *Y*(*t, ν*) is a ccG process, *V*(*t, ν*) becomes a random variable with an exponential distribution for each *t* and *ν*, so its variance equals the square of its expectation:

(17) $$E{\left[V(t,\nu )-EV(t,\nu )\right]}^{2}={\left(EV(t,\nu )\right)}^{2}$$

To obtain a smooth spectrum, measurements are performed many times and the spectrums are accumulated or averaged. Therefore, the Brillouin spectrum obtained by measurements is considered to be expectation *EV*(*t, ν*).

We can calculate the expectation of *V*(*t, ν*) by using the statistical property of Equation (11) as:

(18) $$EV(t,\nu )=\gamma_{\text{R}}L(t,\nu )\overset{t,\nu }{*}\Psi (t,\nu )$$

where $\gamma_{\text{R}}=\upsilon_{\text{g}}\kappa_{2}^{2}Q/2\Gamma^{2}$ is a constant and $\overset{t,\nu }{*}$ denotes a two-dimensional convolution with respect to *t* and *ν*. Functions *L*(*t, ν*) and ψ(*t, ν*) are defined by:

(19) $$L(t,\nu )=\frac{\Gamma^{2}}{\Gamma^{2}+{\left(2\pi (\nu -\nu_{B}(\upsilon_{\text{g}}t/2)\right)}^{2}}$$

(20) $$\Psi (t,\nu )={\left|F_{\tau }[f(\tau )h\left(t-\tau \right)]\right|}^{2}$$

respectively, where 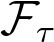 denotes the Fourier transform with respect to *τ*. Equation (18) is derived in Appendix A.

We note that *L*(*t, ν*) is a time-varying Lorentzian spectrum and is determined only by the characteristics of an optical fiber, whereas ψ(*t*, *ν*) depends on the sensing mechanism of BOTDR. Since Equation (18) implies that function, ψ(*t*, *ν*), obscures the details of *L*(*t, ν*), we refer to Ψ(*t*, *ν*) as a point spread function (PSF).

### 2.5. Performance Limit of BOTDR

Though the ideal Brillouin spectrum is the Lorentzian spectrum, the observed Brillouin spectrum is spread by the PSF. Therefore, it is desirable for the PSF to have a shape close to a two-dimensional *δ*-function; that is, narrow in both time and frequency directions. However, by the following uncertainty relation (see Appendix B):

(21) $$2\Delta_{\text{T}}\cdot 2\Delta_{\text{F}}\geq \frac{2}{\pi }$$

the product of time and frequency widths of the PSF cannot be reduced below a certain value. Since the time width corresponds to the spatial resolution, there is a limit, in principle, to improving both spatial and frequency resolution simultaneously through one measurement with whatever pump pulses and low-pass filters are used.

## 3. BOTDR by the Synthetic Approach

To overcome the resolution limitation of conventional BOTDR described in the previous section, a synthetic spectrum approach, which we refer to as synthetic BOTDR (S-BOTDR), is proposed.

### 3.1. Elements of the Point Spread Function

We consider a short pulse, *f*_1_(*t*), and a long pulse, *f*_2_(*t*), as elements of composite pump light:

(22) $$f_{1}(t)=I_{\left[t_{0},t_{0}+D_{1}\right]}(t)$$

(23) $$f_{2}(t)=rI_{\left[0,D_{2}\right]}(t)$$

where *I*_[_*_a,b_*_]_ is the definition function of interval [*a, b*], *D*_1_ and *D*_2_ are the pulse widths of two pulses and *r* is the amplitude ratio of the two pulses. The start time of the short pulse is denoted by *t*_0_.

Similar to the PPP-BOTDA method [8,9], *D*_1_ is set as less than the spatial resolution to be attained and *D*_2_ is taken as sufficiently longer than the acoustic wave lifetime, 2/Γ_B_, say about 9 ns, to improve frequency resolution. A low-pass filter is also composed of two elements, each of which is a matched filter of short or long pulses:

(24) $$h_{1}(t)=f_{1}(-t)$$

(25) $$h_{2}(t)=f_{2}(-t)$$

By using the above elements, a pump light and a low-pass filter are composed as:

(26) $$f_{\theta }(t)=f_{1}(t)+e^{i\theta }f_{2}(t)$$

(27) $$h_{\phi }(t)=h_{1}(t)+e^{i\phi }h_{2}(t)$$

where *θ* and *ϕ* are the phase differences of the two elements. The PSF corresponding to the composite pump light and low-pass filter becomes:

(28) $$\begin{array}{ll} \Psi_{\theta ,\phi }(t,\nu ) & ={\left|F_{\tau }\{f_{\theta }(\tau )h_{\phi }(t-\tau )\}\right|}^{2}\\ & ={\left|F_{11}\right|}^{2}+{\left|F_{12}\right|}^{2}+{\left|F_{21}\right|}^{2}+{\left|F_{22}\right|}^{2}+2ℜ[e^{-i\theta }(F_{11}F_{21}^{*}+F_{12}F_{22}^{*})\\ & +e^{-i\phi }(F_{11}F_{12}^{*}+F_{21}F_{22}^{*})+e^{-i(\theta -\phi )}F_{12}F_{21}^{*}+e^{-i(\theta +\phi )}F_{11}F_{22}^{*}] \end{array}$$

where ℜ[·] denotes the real part, and the RHS is composed of functions:

(29) $$\begin{array}{ll} F_{kl} & \equiv F_{kl}(t,\nu )=F_{\tau }[f_{k}(\tau )h_{l}(t-\tau )]\\ & \begin{array}{ll} =F_{\tau }[f_{k}(\tau )f_{l}(\tau -t)], & k,l=1,2 \end{array} \end{array}$$

**Figure 3.**
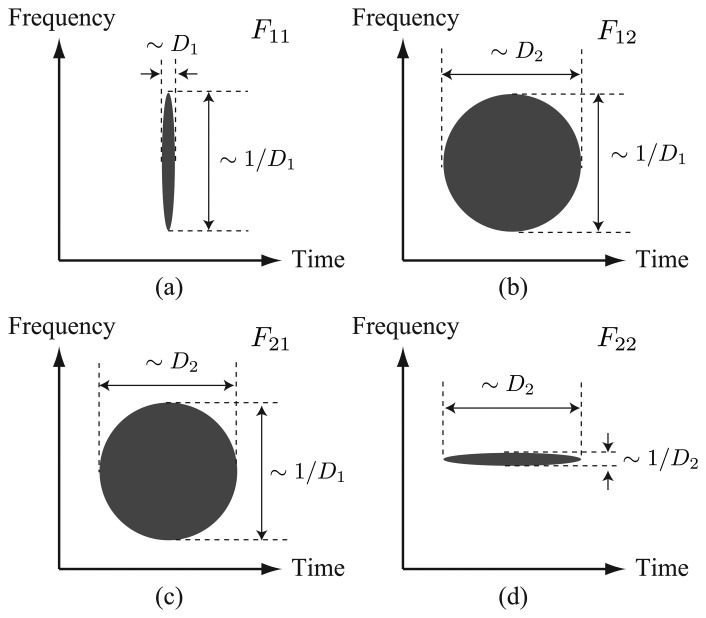
Illustration of *F_kl_, k, l* = 1, 2 spreadness. |*F_kl_*|'s take large values in the black regions: (**a**) *F*_11_; (**b**) *F*_12_; (**c**) *F*_21_; and (**d**) F_22_.

The spreadness of function *F_kl_* has the following features, due to the difference in pulse widths of the two pulses (see also Figure 3):

$$\begin{array}{cc} F_{11}: & \text{small in time and large in frequency},\\ F_{12}\;\text{and}\;F_{21}: & \text{large in both time and frequency},\\ F_{22}: & \text{large in time and small in frequency}. \end{array}$$

Therefore, among the terms of the RHS of Equation (28), only the term, $F_{11}F_{22}^{*}$, has the ideal property that it is localized both in time and frequency, and the other terms are undesired elements for the BOTDR measurement.

### 3.2. PSF by the Synthetic Approach

To extract only element $ℜ(F_{11}F_{22}^{*})$ from the PSF, we prepare *p* phase pairs ((*θ_j_, ϕ_j_*), *j* = 1, 2, ⋯, *p*) and *p* pairs of pump lights and low-pass filters:

(30) $$f^{\left(j\right)}(t)=f_{1}(t)+e^{i\theta_{j}}f_{2}(t)$$

(31) $$\begin{array}{r} h^{\left(j\right)}(t)=h_{1}(t)+e^{i\phi_{j}}h_{2}(t)\\ j=1,2,\cdots ,p \end{array}$$

The Brillouin spectrum and the PSF obtained by using *f*^(^*^j^*^)^(*t*) and *h*^(^*^j^*^)^(*t*) are denoted by *V*^(^*^j^*^)^(*t, ν*) and Ψ^(^*^j^*^)^(*t, ν*), respectively The synthetic spectrum is defined as:

(32) $$V_{\text{S}}(t,\nu )=\sum_{j=1}^{p}c_{j}V^{\left(j\right)}(t,\nu )$$

where *c_j_, j* = 1, 2, ⋯, *p* are weighting coefficients. The corresponding synthetic PSF becomes:

(33) $$\Psi_{\text{S}}(t,\nu )=\sum_{j=1}^{p}c_{j}\Psi^{\left(j\right)}(t,\nu )$$

The *j*-th PSF is represented as:

(34) $$\Psi^{\left(j\right)}(t,\nu )={\left|F_{\tau }[f^{\left(j\right)}(\tau )h^{\left(j\right)}(t-\tau )]\right|}^{2}$$

which is expanded as:

(35) $$\begin{array}{ll} \Psi^{\left(j\right)}(t,\nu )= & {\left|F_{11}\right|}^{2}+{\left|F_{12}\right|}^{2}+{\left|F_{21}\right|}^{2}+{\left|F_{22}\right|}^{2}\\ & +2ℜ[e^{-i\theta_{j}}(F_{11}F_{21}^{*}+F_{12}F_{22}^{*})+e^{-i\phi_{j}}(F_{11}F_{12}^{*}+F_{21}F_{22}^{*})\\ & +e^{-i(\theta_{j}-\phi_{j})}F_{12}F_{21}^{*}+e^{-i(\theta_{j}+\phi_{j})}F_{11}F_{22}^{*} \end{array}$$

If the following relation holds by combining the PSFs, an ideal PSF is obtained.

(36) $$\Psi_{\text{S}}(t,\nu )=2pℜ[F_{11}F_{22}^{*}]$$

This problem is equivalent to finding *θ_j_, ϕ_j_, c_j_, j* = 1, 2, ⋯, *p* that satisfy:

(37) $$\left(\begin{array}{cccc} 1 & 1 & \cdots & 1\\ e^{-i\theta_{1}} & e^{-i\theta_{2}} & \cdots & e^{-i\theta_{p}}\\ e^{-i\phi_{1}} & e^{-i\phi_{2}} & \cdots & e^{-i\phi_{p}}\\ e^{-i(\theta_{1}-\phi_{1})} & e^{-i(\theta_{2}-\phi_{2})} & \cdots & e^{-i(\theta_{p}-\phi_{p})}\\ e^{-i(\theta_{1}+\phi_{1})} & e^{-i(\theta_{2}+\phi_{2})} & \cdots & e^{-i(\theta_{p}+\phi_{p})} \end{array}\right)\;\left(\begin{array}{c} c_{1}\\ c_{2}\\ \vdots \\ c_{p} \end{array}\right)=\left(\begin{array}{l} 0\\ 0\\ 0\\ 0\\ p \end{array}\right)$$

Though the general solution has not been obtained, the following solution with *p* = 4 is at least a solution to Equation (37):

(38) $$\begin{array}{ccc} \left(\begin{array}{c} \theta_{1}\\ \theta_{2}\\ \theta_{3}\\ \theta_{4} \end{array}\right)=\left(\begin{array}{c} 0\\ \pi /2\\ \pi \\ 3\pi /2 \end{array}\right), & \left(\begin{array}{l} \phi_{1}\\ \phi_{2}\\ \phi_{3}\\ \phi_{4} \end{array}\right)=\left(\begin{array}{c} 0\\ 3\pi /2\\ 0\\ \pi /2 \end{array}\right), & \left(\begin{array}{c} c_{1}\\ c_{2}\\ c_{3}\\ c_{4} \end{array}\right) \end{array}=\left(\begin{array}{c} -1\\ 1\\ -1\\ 1 \end{array}\right)$$

Hereafter, we use this solution.

The PSF by the synthetic approach is represented as:

(39) $$\Psi_{S}(t,\nu )=8ℜ[F_{11}F_{22}^{*}]$$

and is concentrated around the origin in both the time and frequency directions, as shown in the previous section. However, in addition to this, its two-dimensional integral in the time-frequency space must be a certain positive value to approximate a two-dimensional *δ*-function. The two-dimensional integral of the synthetic PSF of Equation (39) becomes:

(40) $$\int_{-\infty }^{\infty }\int_{-\infty }^{\infty }\Psi_{\text{S}}(t,\nu )d\nu dt=8{\left(\int_{-\infty }^{\infty }f_{1}(t)f_{2}(t)dt\right)}^{2}$$

If two pulses are separated in time, this integral becomes zero, and if they overlap partially, the integral is proportional to the width of the overlapping parts. To maximize the integral, the short pulse must be included in the long pulse, and the integral becomes $8r^{2}D_{1}^{2}$.

For two pulses of Equations (22) and (23), the synthetic PSF becomes:

(41) $$\Psi_{S}(t,\nu )=8r^{2}\cos\pi (D_{2}-D_{1}-2t_{0})\nu \frac{\sin\pi (D_{1}-|t|)\nu }{\pi \nu }\frac{\sin\pi (D_{2}-|t|)\nu }{\pi \nu }I_{\left[-D_{1},D_{1}\right]}(t)$$

If *D*_1_ is sufficiently small and *D*_2_ is sufficiently large, the PSF is approximated as:

(42) $$\Psi_{\text{S}}(t,\nu )\approx 8r^{2}D_{1}^{2}\delta (\nu )$$

and the synthetic spectrum is approximated as:

(43) $$EV_{S}(t,\nu )\approx 8r^{2}D_{1}^{2}\gamma_{R}L(t,\nu )$$

**Figure 4.**
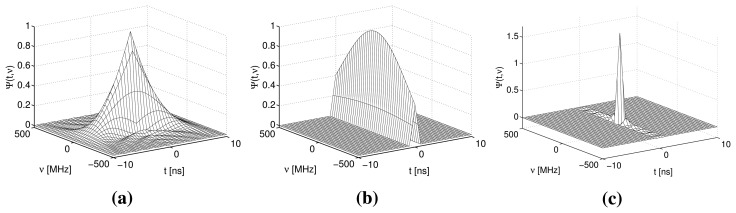
Point spread functions: (**a**) conventional BOTDR (*D* = 10 ns); (**b**) conventional BOTDR (*D* = 1 ns); (**c**) synthetic (S)-BOTDR (*D*_1_ = 1 ns; and *D*_2_ = 50 ns).

**Figure 5.**
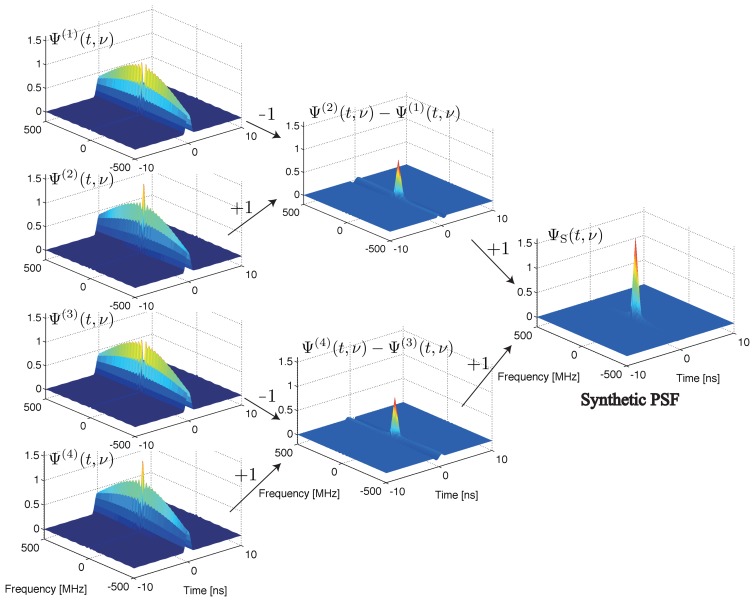
Composition of the synthetic point spread function of S-BOTDR.

The PSFs for a conventional BOTDR and S-BOTDR are plotted in Figure 4. While the PSF of the conventional BOTDR spreads in either the time or frequency direction, that of S-BOTDR is concentrated at the origin and approximates a *δ*-function. To clarify the mechanism of generating such a *δ*-like function, the composition of the synthetic PSF is shown in Figure 5. Although each of the PSF, Ψ^(^*^j^*^)^, *j* = 1, 2, 3, 4, concentrates in the time direction, it is spread in the frequency direction. This spreading is partially suppressed with two-by-two combination, Ψ^(2)^ − Ψ^(1)^ and Ψ^(4)^ − Ψ^(3)^, and is completely suppressed with the use of all PFS elements.

### 3.3. S-BOTDR System

**Figure 6.**
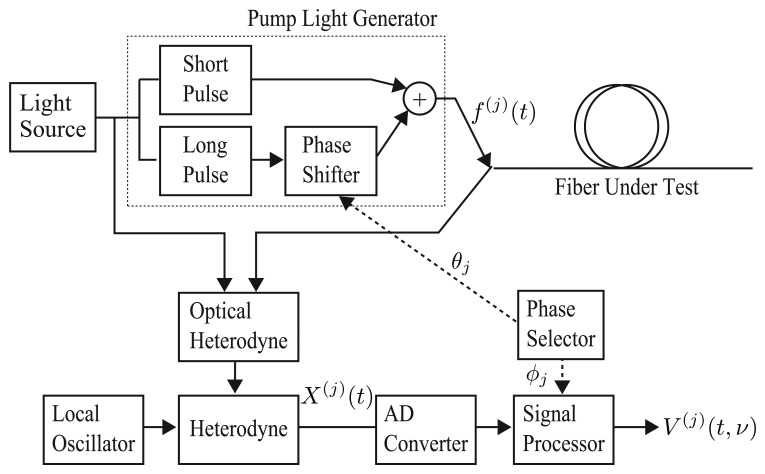
Block diagram of S-BOTDR.

**Figure 7.**
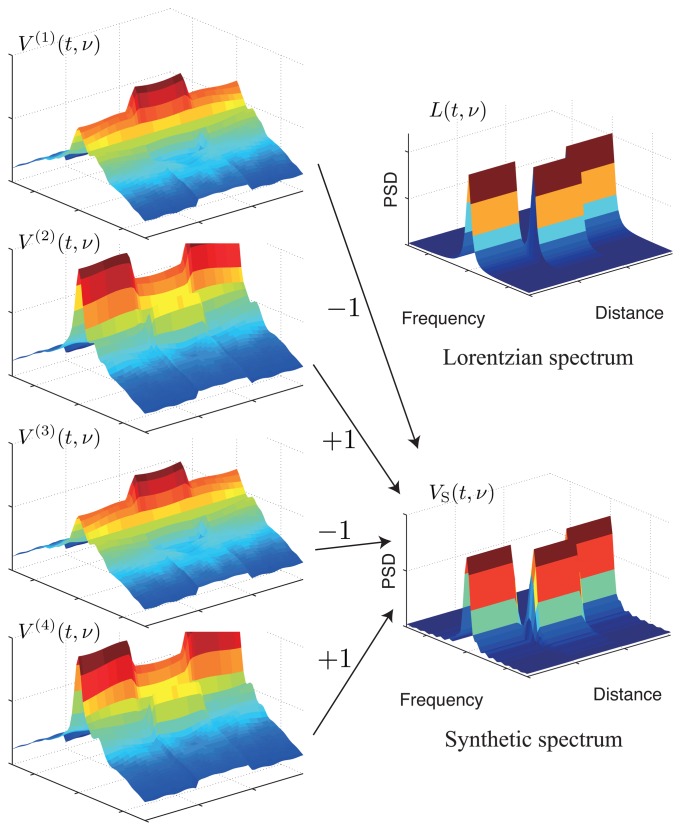
Composition of the synthetic spectrum of S-BOTDR.

As shown in the analysis of the previous section, the synthetic Brillouin spectrum has the ideal property of being close to a Lorentzian spectrum. An S-BOTDR system for obtaining the synthetic Brillouin spectrum is constructed as shown in Figure 6. The composition of the synthetic Brillouin spectrum is followed by Equation (32) and is illustrated in Figure 7. Although the spectrum of each measurement exhibits high spatial resolution, it is considerably different from the Lorentzian spectrum. Only the combination of all four spectrums produces the Lorentzian spectrum.

The ideal property described in the previous section is for the expectation of the synthetic Brillouin spectrum. Achieving this property requires averaging or summing of the spectrums obtained by a number of measurements.

### 3.4. Parameter Selection for S-BOTDR

The pump lights of S-BOTDR are composed of short and long pulses and include parameters *t*_0_ and *r*: *t*_0_ is the difference of pulse start times and *r* is the amplitude ratio of the two pulses. These parameters affect the performance of S-BOTDR.

**Selection of** *t*_0_ If the short pulse is included in the long pulse, *i.e.*, if 0 ≤ *t*_0_ ≤ *D_2_* − *D*_1_, the integral of Equation (40) takes the maximum value. In addition, the value of *t*_0_ affects the concentration of the PSF near the origin. The degree of concentration is estimated by the integral:

(44) $$C(\nu ;t_{0})\int_{-\nu }^{\nu }\int_{-\infty }^{\infty }\Psi (t,\nu^{\prime })\text{dtd}\nu^{\prime }/(8r^{2}D_{1}^{2})$$

If *C*(*ν*; *t*_0_) approaches one for small *ν*, the PSF is considered to be concentrated near the origin of the frequency axis.

**Figure 8.**
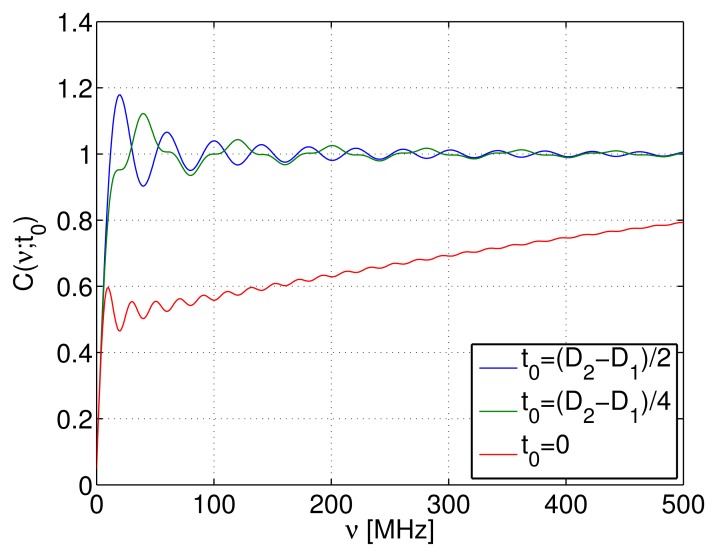
*C*(*ν; t*_0_): the integral of the point spread function (PSF).

The values of *C*(*ν*; *t*_0_) are plotted for different *t*_0_'s in Figure 8. Here, for *t*_0_ = (*D*_2_ − *D*_1_)/2, the short pulse is located at the center of the long pulse; for *t*_0_ = 0, the short pulse is at the edge of the long pulse, and the medium case is *t*_0_ = (*D*_2_ − *D*_1_)/4. From Figure 8, we find that when the short pulse is located at the edge of the long pulse, the degree of concentration is reduced. Thus, we adopt *t*_0_ = (*D*_2_ − *D*_1_)/2.

**Selection of** *r* It is desirable for S-BOTDR to suppress the synthetic spectrum's fluctuation as much as possible while keeping the peak level high. To meet these requirements, we define the signal-to-fluctuation ratio (SFR) as:

(45) $$\text{SFR}=\frac{\mu_{\text{peak}}^{2}}{\sigma_{\text{peak}}^{2}}$$

where *μ*_peak_ and $\sigma_{\text{peak}}^{2}$ are the mean and the variance of the synthetic spectrum at the peak, respectively As shown in Appendix C, the SFR is represented explicitly as a function of *r*, and the optimal *r*, which maximizes the SFR, is given by:

(46) $$r_{\text{opt}}={\left(\frac{{\left(\Gamma D_{1}\right)}^{2}-2\Gamma D_{1}+2(1-e^{-\Gamma D_{1}})}{{\left(\Gamma D_{2}\right)}^{2}-2\Gamma D_{2}+2(1-e^{-\Gamma D_{2}})}\right)}^{1/4}$$

Numerical examples of *r*_opt_ are shown in Table 1. We used the optimal *r* in simulations and experiments.

**Figure 9.**
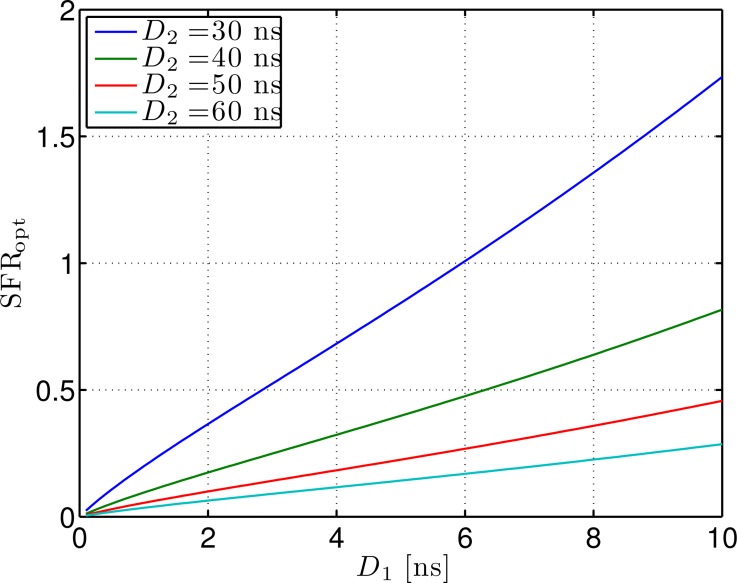
Signal-to-fluctuation ratio for various *D*_1_ and *D*_2_.

The SFR of S-BOTDR is obtained by substituting (46) into (76) in Appendix C. The SFR values for various *D*_1_ and *D*_2_ are plotted in Figure 9. From this figure, we find that SFR is nearly proportional to *D*_1_ for fixed *D*_2_. On the other hand, BFS estimation error variance is inversely proportional to SFR [20]. Therefore, the BFS estimation error variance is nearly proportional to 1/D_1_.

## 4. Evaluation by Simulation

**Figure 10.**
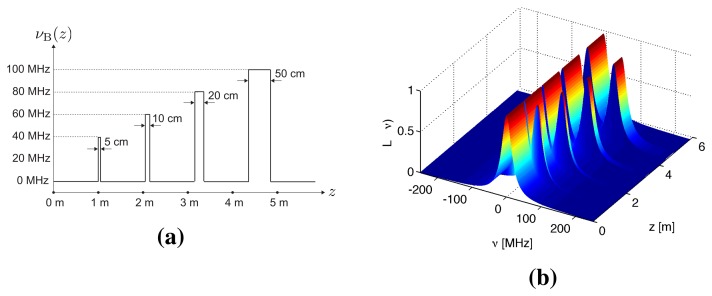
Simulation condition: (**a**) assumed Brillouin frequency shift (BFS) and (**b**) the Lorentzian spectrum.

**Figure 11.**
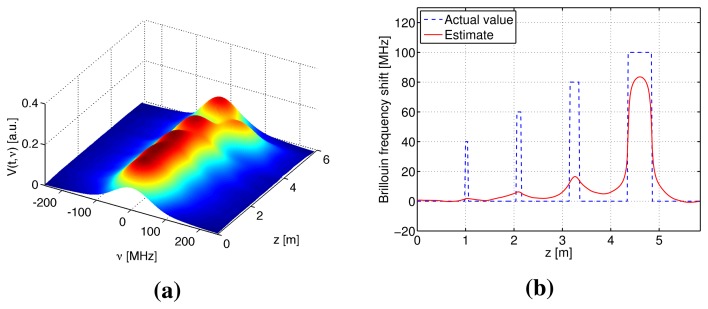
Simulation results of conventional BOTDR with D = 10 ns: (**a**) the Brillouin spectrum and (**b**) the estimate of BFS.

**Figure 12.**
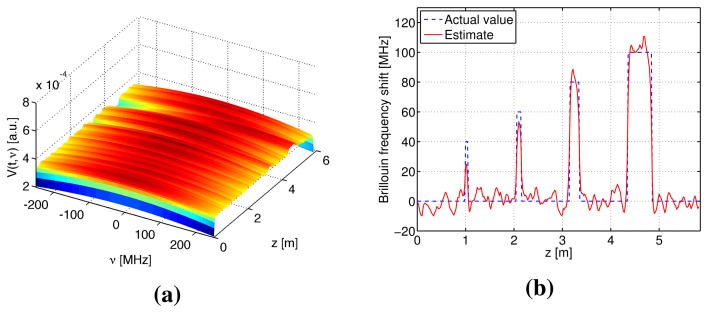
Simulation results of conventional BOTDR with *D* = 1 ns: (**a**) the Brillouin spectrum and (**b**) the estimate of BFS.

**Figure 13.**
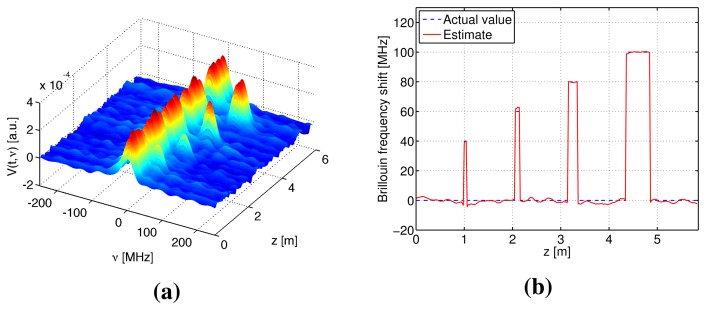
Simulation results of S-BOTDR with *D*_1_ = 1 ns and *D*_2_ = 50 ns: (**a**) the composite Brillouin spectrum and (**b**) the estimate of BFS.

Numerical simulations were performed to verify the S-BOTDR and compare it with the conventional BOTDR. The condition of the optical fiber is shown in Figure 10. In the figure, an assumed BFS is shown in (a) and the corresponding Lorentzian spectrum is shown in (b). Simulation results are shown in Figures 11, 12, and 13. The parameter values for processing are as follows:

- Conventional BOTDR (Figures 11 and 12)
  Pulse width is *D* = 10 and 1 ns.
- S-BOTDR (Figure 13)
  Pulse widths are *D*_1_ = 1 ns for a short pulse and *D*_2_ = 50 ns for a long pulse. The amplitude ratio is r = 0.066. The short pulse is located at the center of the long pulse.

All results were obtained by summing 2^12^ trials. For conventional BOTDR with a 10-ns pulse in Figure 11, the linewidth of the spectrum was small and smooth; however, spatial resolution was inferior and could not detect any interval less than 50 cm. For the conventional BOTDR with a 1-ns pulse in Figure 12, because the linewidth of the spectrum was quite large (about 1 GHz), the estimation accuracy of the BFS was inferior. In the case of the S-BOTDR in Figure 13, the Brillouin spectrum was close to the Lorentzian spectrum; hence, accurate BFS estimates were obtained. Thus, S-BOTDR has a high-resolution capability exceeding that of conventional BOTDR.

The simulation was performed using the exact values of the input parameters. In an actual situation, these parameters are, however, subject to errors. As in S-BOTDR four measurements are combined, the input light amplitude fluctuation and phase-setting errors become issues of concern. Among them, the light amplitude fluctuation becomes negligible by averaging many replicate measurements. The errors in the phase differences between short and long elements are, on the other hand, anticipated. We performed a series of additional simulations in which the phase differences were subject to random errors. The results clearly demonstrated that they did not affect the spatial resolution, while the BFS estimation error increased only slightly, even if phase errors as high as 40 degrees were applied.

## 5. Experimental Verification

**Figure 14.**
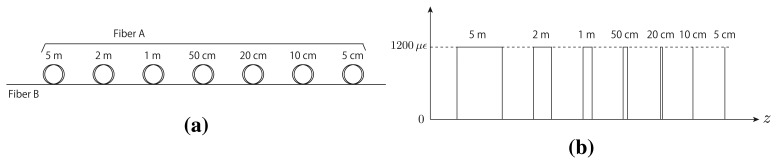
The fiber under testing used in experiment: (**a**) the fiber setup and (**b**) the equivalent strain.

**Figure 15.**
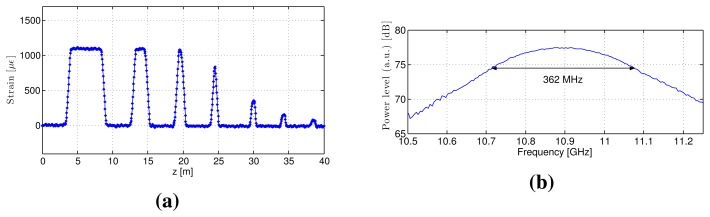
Experimental BOTDR results: (**a**) strain estimates and (**b**) the Brillouin spectrum at the 5-m strain interval.

We performed BOTDR and S-BOTDR experiments using a test fiber. We constructed a test fiber by connecting two kinds of fibers: A and B (Figure 14a). The BFS between fibers A and B differs, and the difference corresponds to a strain of 1,200 μ∊. In the experiments, fibers A and B are considered strained fibers and strain-free fibers, respectively. The replicate measurement numbers were 2^16^ in all cases. The experimental result for BOTDR is shown in Figure 15. The pulse width of the pump light for BOTDR was 5 ns. Strain estimates and the Brillouin spectrum at the 5-m strain interval are plotted in Figure 15a,b, respectively. The linewidth of the Brillouin spectrum exceeded 360 MHz (Figure 15b). Although intervals larger than or equal to one meter were accurately detected, the strain estimates of the other intervals differed from the true values (Figure 15a).

We performed S-BOTDR experiments using the following combination of short and long elements for the pump light and the low-pass filter:

**Case 1** *D*_1_ = 7.8 ns, *D*_2_ = 60 ns, *r* = 0.26
**Case 2** *D*_1_ = 2.7 ns, *D*_2_ = 50 ns, *r* = 0.14
**Case 3** *D*_1_ = 1.6 ns, *D*_2_ = 50 ns, *r* = 0.093

**Figure 16.**
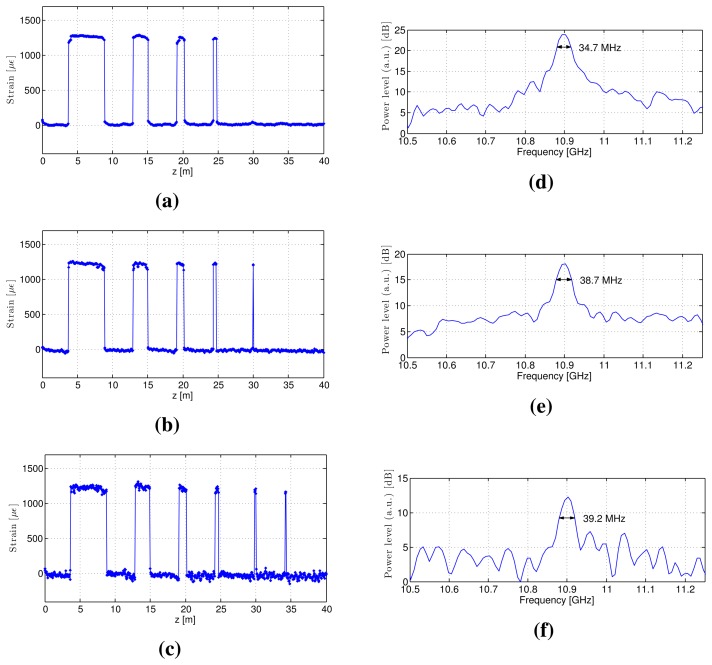
Experimental S-BOTDR results: (**a–c**) Strain estimates of Cases 1 to 3; (**d–f**) composite Brillouin spectrums at the 5-m strain interval of Cases 1 to 3.

Here, the values of *r* were determined to be the optimal values. The strain estimates in each case are plotted in Figure 16a–c. We attained a spatial resolution of 50 cm in Case 1, 20 cm in Case 2 and 10 cm in Case 3. Figures 16a-c also indicate that the fluctuation of estimates increases as *D*_1_ becomes small. This is because the estimation error variance is nearly proportional to 1/*D*_1_, as is described in Section 3.4. Therefore, some compromise is required to select small *D*_1_. The synthetic spectrums at the 5-m strain interval are shown in Figure 16d,e. In all cases, their linewidths were less than 40 MHz. This is because the linewidth of the synthetic spectrums is determined by the reciprocal of the long pulse's length. These experimental results prove that we attained 10-cm spatial resolution that exceeds the limit of conventional BOTDR.

## 6. Conclusions

We showed that conventional BOTDR has, in principle, a resolution limit and proposed a synthetic spectrum approach, referred to as S-BOTDR, to overcome this limit. S-BOTDR uses composite pump lights and low-pass filters that are composed of short and long elements with different phases. Four BOTDR measurements are performed using four specific pairs of phases. By combining four Brillouin spectrums with specific weights, an ideal spectrum is obtained. The spectrum is close to the Lorentzian spectrum, and the resolution limit, in principle, disappears. We experimentally evaluated S-BOTDR and proved that we attained a 10-cm resolution that exceeds the resolution limit of conventional BOTDR.

## Figures and Tables

**Figure 2.**
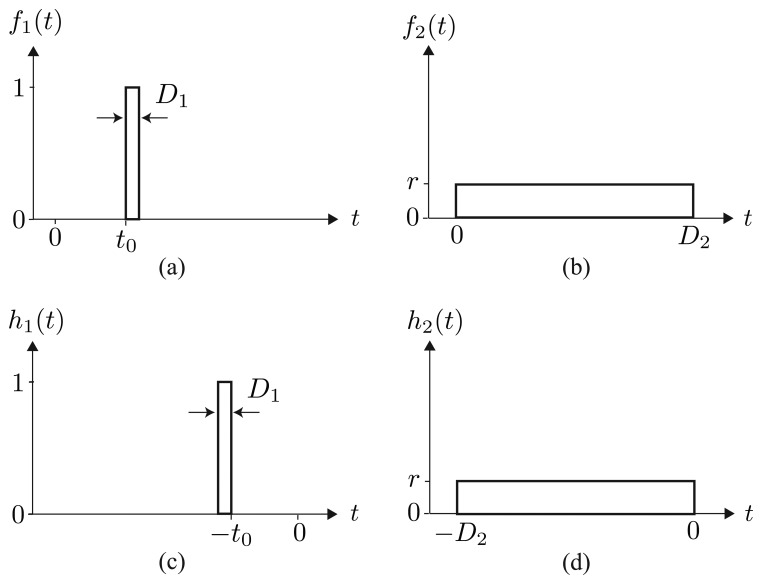
Pump lights and matched filters: (**a**) Element 1 of the pump light; (**b**) Element 2 of the pump light; (**c**) Element 1 of the low-pass filter; and (**d**) Element 2 of the low-pass filter.

**Table 1.** Numerical examples of *r*_opt_.

*D*_1_ [ns]	*D*_2_ (ns)			

	**30**	**40**	**50**	**60**
1	0.0896	0.0750	0.0656	0.0589
2	0.1499	0.1254	0.1096	0.0985
3	0.2020	0.1690	0.1477	0.1327
4	0.2493	0.2085	0.1823	0.1638
5	0.2930	0.2451	0.2143	0.1925
6	0.3342	0.2795	0.2444	0.2195
7	0.3731	0.3121	0.2728	0.2451
8	0.4102	0.3431	0.3000	0.2695
9	0.4458	0.3728	0.3260	0.2929
10	0.4800	0.4014	0.3510	0.3153

## Appendix A. Derivation of (18)

In this appendix, we derive Equation (18), which is the convolutional representation of the expectation of the Brillouin spectrum. Substituting Equation (15) into Equation (16), we have:

(47) $$\begin{array}{ll} V(t,\nu )= & \kappa_{2}^{2}\int_{-\infty }^{\infty }\int_{-\infty }^{\infty }h(t-\tau )h*(t-\tau^{\prime })e^{-2\pi i\nu (\tau -\tau^{\prime })}\\ & \times \int_{0}^{L_{\text{f}}}\int_{0}^{L_{\text{f}}}f\left(\tau -\frac{2z}{\upsilon_{g}}\right)f*\left(\tau^{\prime }-\frac{2z^{\prime }}{\upsilon_{g}}\right)\zeta *(z,\tau )\zeta (z^{\prime },\tau^{\prime })\text{dzd}z^{\prime }d\tau d\tau^{\prime } \end{array}$$

whose expectation is calculated using Equation (14), such that:

(48) $$\begin{array}{ll} EV(t,\nu )= & \frac{\kappa_{2}^{2}Q}{2\Gamma }\int_{-\infty }^{\infty }\int_{-\infty }^{\infty }h(t-\tau )h*(t-\tau^{\prime })e^{-2\pi i\nu (\tau -\tau^{\prime })}\\ & \times \int_{0}^{L_{\text{f}}}f\left(\tau -\frac{2z}{\upsilon_{g}}\right)f*\left(\tau^{\prime }-\frac{2z}{\upsilon_{g}}\right)e^{2\pi i\nu_{\text{B}}(z)(\tau -\tau^{\prime })}e^{-\Gamma |\tau -\tau^{\prime }|}\text{dzd}\tau d\tau^{\prime } \end{array}$$

We assume that functions that contain *ν*_B_(*z*) vanish outside the interval [0, *L*_f_]. The integral interval can then be replaced by [−∞, ∞], and we have:

(49) $$\begin{array}{ll} EV(t,\nu ) & =\frac{\upsilon_{g}\kappa_{2}^{2}Q}{4\Gamma }\int_{-\infty }^{\infty }\int_{-\infty }^{\infty }\int_{-\infty }^{\infty }f(\tau -s)h(t-\tau )\\ & \times f*(\tau^{\prime }-s)h*(t-\tau^{\prime })e^{-2\pi i(\nu -\nu_{B}(\upsilon_{g}s/2))(\tau -\tau^{\prime })}e^{-\Gamma |\tau -\tau^{\prime }|}\text{dsd}\tau d\tau^{\prime }\\ & =\gamma_{R}\int_{-\infty }^{\infty }\int_{-\infty }^{\infty }\int_{-\infty }^{\infty }f(\tau -s)h(t-\tau )\\ & \times f*(\tau -\tau^{\prime \prime }-s)h*(t-\tau +\tau^{\prime \prime })d\tau \frac{\Gamma }{2}e^{-2\pi i(\nu -\nu_{B}(\upsilon_{g}s/2))\tau^{\prime \prime }}e^{-\Gamma |\tau \prime \prime |}\text{dsd}\tau \prime \prime \\ & =\gamma_{R}\int_{-\infty }^{\infty }e^{-2\pi i\nu \tau^{\prime \prime }}\int_{-\infty }^{\infty }\left\{[f(\tau^{\prime \prime }-s)h(t-\tau^{\prime \prime })\right]\\ & \overset{\tau^{\prime \prime }}{*}[f*(-\tau^{\prime \prime }-s)h*(t+\tau^{\prime \prime })]\}\frac{\Gamma }{2}e^{2\pi i\nu_{\text{B}}(\upsilon_{g}s/2)\tau^{\prime \prime }}e^{-\Gamma |\tau^{\prime \prime }|}\text{dsd}\tau^{\prime \prime } \end{array}$$

where we set $\gamma_{\text{R}}=\upsilon_{\text{g}}\kappa_{2}^{2}Q/2\Gamma^{2}$. Since the Fourier transform of a product is the convolution of the corresponding transforms, we have:

(50) $$\begin{array}{ll} EV(t,\nu ) & =\gamma_{R}\int_{-\infty }^{\infty }\{F_{\tau^{\prime \prime }}[f(\tau^{\prime \prime }-s)h(t-\tau^{\prime \prime })]F_{\tau^{\prime \prime }}[f*(-\tau^{\prime \prime }-s)h*(t+\tau^{\prime \prime })]\}\overset{\nu }{*}L(s,v)ds\\ & =\gamma_{R}\int_{-\infty }^{\infty }{\left|F_{\tau^{\prime \prime }}[f(\tau^{\prime \prime }-s)h(t-\tau^{\prime \prime })]\right|}^{2}\overset{\nu }{*}L(s,v)ds\\ & =\gamma_{R}\int_{-\infty }^{\infty }{\left|F_{\tau }[f(\tau )h(t-s-\tau )]\right|}^{2}\overset{\nu }{*}L(s,v)ds\\ & =\gamma_{R}{\left|F_{\tau }[f(\tau )h(t-\tau )]\right|}^{2}\overset{t,\nu }{*}L(s,v) \end{array}$$

where *L*(*t, ν*) is defined by Equation (19) and is the Fourier transform of (Γ/2) exp(2*πiν*_B_(*υ*_g_*t*/2)*τ* − Γ|*τ*|) with respect to *τ*. Moreover, defining Ψ(*t, ν*) by Equation (20) yields Equation (18).

## Appendix B. Uncertainty Relation of PSF

The frequency integral of the PSF Equation (20) gives a temporal PSF as follows:

(51) $$\Psi_{T}(t)\equiv \int_{-\infty }^{\infty }\Psi (t,\nu )d\nu =|f(t){|}^{2}*|h(t){|}^{2}$$

Center *μ*_T_ and radius Δ_T_ of Ψ_T_(*t*) are obtained as:

(52) $$\mu_{\text{T}}\overset{\text{def}}{=}\frac{1}{N}\int_{-\infty }^{\infty }t\Psi_{\text{T}}(t)dt=\mu_{f}+\mu_{h}$$

(53) $$\Delta_{\text{T}}^{2}\overset{\text{def}}{=}\frac{1}{N}\int_{-\infty }^{\infty }{\left(t-\mu_{T}\right)}^{2}\Psi_{\text{T}}(t)dt=\sigma_{f}^{2}+\sigma_{h}^{2}$$

where:

(54) $$N=\int_{-\infty }^{\infty }\Psi_{\text{T}}(t)dt$$

is a normalizing constant and:

(55) $$\mu_{f}=\frac{1}{{\left\|f\right\|}^{2}}\int_{-\infty }^{\infty }t{\left|f(t)\right|}^{2}dt$$

(56) $$\sigma_{f}^{2}=\frac{1}{{\left\|f\right\|}^{2}}\int_{-\infty }^{\infty }{\left(t-\mu_{f}\right)}^{2}{\left|f(t)\right|}^{2}dt$$

Here, ‖ · ‖ denotes a *L*^2^ norm, and *μ_h_* and *σ_h_* are defined in the same way. Similarly, a frequency PSF is obtained by a time integral as follows:

(57) $$\Psi_{\text{F}}(\nu )\equiv \int_{-\infty }^{\infty }\Psi (t,\nu )dt=\int_{-\infty }^{\infty }{\left|\hat{f}(\nu +\xi )\right|}^{2}\cdot {\left|\hat{h}(\xi )\right|}^{2}d\xi$$

where *f̂* and *ĥ* are the Fourier images of *f* and *h*, respectively A center and a radius of Ψ_F_(*ν*) are also obtained as:

(58) $$\begin{array}{ll} \mu_{\text{F}}=\mu_{\hat{f}}-\mu_{\hat{h}}, & \Delta_{\text{F}}^{2}=\sigma_{\hat{f}}^{2} \end{array}+\sigma_{\hat{h}}^{2}$$

where *μ_f̂_*, *μ_ĥ_, σ_f̂_* and *σ_ĥ_* are defined the same as *μ_f_* and *σ_f_*. As is well known in Fourier analysis, the following uncertainty relations hold:

(59) $$\begin{array}{ll} \sigma_{f}\sigma_{\hat{f}}\geq \frac{1}{4\pi }, & \sigma_{h}\sigma_{\hat{h}}\geq \frac{1}{4\pi } \end{array}$$

the use of which, we have:

(60) $$\Delta_{\text{T}}^{2}\Delta_{\text{F}}^{2}=(\sigma_{f}^{2}+\sigma_{h}^{2})(\sigma_{\hat{f}}^{2}+\sigma_{\hat{h}}^{2})\geq \frac{2}{{\left(4\pi \right)}^{2}}+\frac{1}{{\left(4\pi \right)}^{2}}\left(\frac{\sigma_{f}^{2}}{\sigma_{h}^{2}}+\frac{\sigma_{h}^{2}}{\sigma_{f}^{2}}\right)$$

Since $\sigma_{f}^{2}/\sigma_{h}^{2}+\sigma_{h}^{2}/\sigma_{f}^{2}$ in the last term takes the minimum value of two, when *σ_f_* = *σ_h_*, we obtain:

(61) $$\Delta_{\text{T}}^{2}\Delta_{\text{F}}^{2}\geq \frac{1}{{\left(2\pi \right)}^{2}}$$

## Appendix C. Signal-to-Fluctuation Ratio of S-BOTDR

The synthetic spectrum of S-BOTDR *V*_s_(*t, ν*) is the weighted sum of elements *V*^(^*^j^*^)^(*t,ν), j* = 1, 2, 3, 4, as shown in Equation (32). Since all elements are statistically independent and |*c_j_*| = 1, the variance of *V*_s_(*t, ν*) becomes the sum of the variances of *V*^(^*^j^*^)^(*t, ν*), *j* = 1, 2, 3, 4. Moreover, since each *V*^(^*^j^*^)^(*t, ν*) follows an exponential distribution, we have:

(62) $$E{\left[V_{\text{S}}(t,\nu )-EV_{\text{S}}(t,\nu )\right]}^{2}=\sum_{j=1}^{4}{\left(EV^{\left(j\right)}(t,\nu )\right)}^{2}$$

We now arbitrarily fix time *t* and obtain the fluctuation at the peak of the spectrum. For simplicity, we assume that the BFS is constant and *ν*_B_(*υ*_g_*t*/2) = 0. The peak position is then given by *ν* = 0. The expectation of all elements of the synthetic spectrum in S-BOTDR at *ν* = 0 is then given by:

(63) $$EV^{\left(j\right)}(t,0)=\gamma_{\text{R}}\int_{-\infty }^{\infty }\left[L(t,-\nu )\overset{t}{*}\Psi^{\left(j\right)}(t,\nu )\right]d\nu$$

Using the assumption for the BFS, the Lorentzian spectrum can be written as:

(64) $$L(t,\nu )=L_{0}(\nu )\overset{\text{def}}{=}\frac{\Gamma^{2}}{\Gamma^{2}+{\left(2\pi \nu \right)}^{2}}$$

which, substituting into Equation (63), yields:

(65) $$EV^{\left(j\right)}=\gamma_{R}\int_{-\infty }^{\infty }L_{0}(\nu )\int_{-\infty }^{\infty }\Psi^{\left(j\right)}(t,\nu )\text{dtd}\nu$$

where we abbreviate the arguments, because *EV*^(^*^j^*^)^ (*t*, 0) does not depend on *t*. Noting that:

(66) $$L_{0}(\nu )=F_{\tau }\left[\frac{\Gamma }{2}e^{-\Gamma |\tau |}\right]$$

and using Plancherel's theorem and Equation (34), we obtain:

(67) $$\begin{array}{ll} EV^{\left(j\right)} & =\int_{-\infty }^{\infty }F_{\nu }^{-1}[L_{0}(\nu )]F_{\nu }^{-1}\left[\int_{-\infty }^{\infty }\Psi^{\left(j\right)}(t,\nu )dt\right]d\tau \\ & =\gamma_{\text{R}}\int_{-\infty }^{\infty }\frac{\Gamma }{2}e^{-\Gamma |\tau |}\int_{-\infty }^{\infty }[h^{\left(j\right)}(t)h^{\left(j\right)}*(t+\tau )]dt\int_{-\infty }^{\infty }\left[f^{\left(j\right)}(t)f^{(j)*}(t-\tau )\right]\text{dtd}\tau \\ & =\frac{\gamma_{R}\Gamma }{2}\int_{-\infty }^{\infty }e^{-\Gamma |\tau |}(K_{11}+re^{-i\theta_{j}}K_{12}+re^{i\theta_{j}}K_{21}+r^{2}K_{22})\\ & \times (K_{11}+re^{-i\phi_{j}}K_{12}+re^{-i\phi_{j}}K_{21}+r^{2}K_{22})d\tau \end{array}$$

where:

(68) $$\begin{array}{ll} K_{kl}=\int_{-\infty }^{\infty }f_{k}(t)f_{l}(t-\tau )dt=\int_{-\infty }^{\infty }h_{k}(t)h_{l}(t+\tau )dt, & k,l=1,2 \end{array}$$

Substituting the values of Equation (38) into Equation (67), we have:

(69) $$EV^{\left(j\right)}=\frac{\gamma_{\text{R}}\Gamma }{2}(a_{0}+a_{1,j}r^{2}+a_{2}r^{4})$$

where *r* is the amplitude ratio of the short and long pulses appearing in Equation (23), and:

(70) $$a_{0}=\int_{-\infty }^{\infty }e^{-\Gamma |\tau |}K_{11}^{2}d\tau$$

(71) $$a_{1,j}=2\int_{-\infty }^{\infty }e^{-\Gamma |\tau |}(K_{11}K_{22}+\cos(\theta_{j}+\phi_{j})K_{12}^{2}-K_{12}K_{21})d\tau$$

(72) $$a_{2}=\int_{-\infty }^{\infty }e^{-\Gamma |\tau |}K_{22}^{2}d\tau$$

From the relation of Equation (62), $\sigma_{\text{peak}}^{2}$, the variance of the spectrum at the peak, is represented as:

(73) $$\sigma_{\text{peak}}^{2}=\sum_{j=1}^{4}{\left(EV^{\left(j\right)}\right)}^{2}$$

into which substituting Equation (63) gives:

(74) $$\sigma_{\text{peak}}^{2}={\left(\frac{\gamma_{\text{R}}\Gamma }{2}\right)}^{2}\left[4{\left(a_{0}+a_{1}r^{2}+a_{2}r^{4}\right)}^{2}+\sum_{j=1}^{4}(a_{1,j}^{2}-a_{1}^{2})r^{4}\right]$$

where we set $a_{1}=\sum_{j=1}^{4}a_{1,j}/4$.

On the other hand, *μ*_peak_, the mean of the spectrum at the peak, is approximated by Equation (43) as:

(75) $$\mu_{\text{peak}}=\max EV_{\text{S}}(t,\nu )\approx 8r^{2}D_{1}^{2}\gamma_{\text{R}}$$

Substituting Equations (75) and (74) into Equation (45), we have:

(76) $$\text{SFR}=\frac{{\left(8D_{1}^{2}/\Gamma \right)}^{2}}{{\left(\frac{a_{0}}{r^{2}}+a_{1}+a_{2}r^{2}\right)}^{2}+\frac{1}{4}\overset{4}{\underset{j=1}{\text{∑}}}\left(a_{1,j}^{2}-a_{1}^{2}\right)}$$

## Appendix D. Optimal Amplitude Ratio between Short and Long Pulses

The maximization of the SFR of Equation (76) is equivalent to the minimization of:

(77) $$J=\frac{a_{0}}{r^{2}}+a_{1}+a_{2}r^{2}$$

The value of *r* that minimizes *J* is given by:

(78) $$r_{\text{opt}}={\left(\frac{a_{0}}{a_{2}}\right)}^{1/4}$$

which maximizes SFR. From Equations (22), (23) and (68),

(79) $$k_{11}(\tau )=\{\begin{array}{ll} D_{1}-\left|\tau \right|, & \left|\tau \right|\leq D_{1}\\ 0, & \text{otherwise} \end{array}$$

(80) $$k_{22}(\tau )=\{\begin{array}{ll} D_{2}-\left|\tau \right|, & \left|\tau \right|\leq D_{2}\\ 0, & \text{otherwise} \end{array}$$

holds, and by substituting them into Equations (70) and (72), we have:

(81) $$a_{0}=\frac{2}{\Gamma^{3}}[{\left(\Gamma^{2}D_{1}\right)}^{2}-2\Gamma D_{1}+2(1-e^{-\Gamma D_{1}})]$$

(82) $$a_{2}=\frac{2}{\Gamma^{3}}[{\left(\Gamma^{2}D_{2}\right)}^{2}-2\Gamma D_{2}+2(1-e^{-\Gamma D_{2}})]$$

Therefore,

(83) $$r_{\text{opt}}={\left(\frac{{\left(\Gamma D_{1}\right)}^{2}-2\Gamma D_{1}+2(1-e^{-\Gamma D_{1}})}{{\left(\Gamma D_{2}\right)}^{2}-2\Gamma D_{2}+2(1-e^{-\Gamma D_{2}})}\right)}^{1/4}$$

becomes the optimal value of *r*.
